# IL 15 enhances preclinical efficacy of anti-core 1 O-glycans monoclonal antibody NEO-201 against human endometrial and ovarian cancer

**DOI:** 10.3389/fimmu.2026.1652596

**Published:** 2026-02-24

**Authors:** Jamie Hur, Massimo Fantini, Lidia Hernandez, Soumya Korrapati, Elijah F. Edmondson, Maggie Cam, Mayank Tandon, Christopher B. Cole, Kwong Y. Tsang, Philip M. Arlen, Christina M. Annunziata, Maria Pia Morelli

**Affiliations:** 1 Women’s Malignancies Branch, Center for Cancer Research, National Cancer Institute, National Institutes of Health, Bethesda, MD, United States; 2 Precision Biologics, Inc., Bethesda, MD, United States; 3 Molecular Histopathology Laboratory, Laboratory Animal Sciences Program, Frederick National Laboratory for Cancer Research, Frederick, MD, United States; 4 Center for Cancer Research Collaborative Bioinformatics Resource (CCBR), National Cancer Institute, National Institutes of Health, Bethesda, MD, United States; 5 Advanced Biomedical Computational Science, Frederick National Laboratory for Cancer Research, Frederick, MD, United States; 6 Department of Gastrointestinal Medical Oncology, The University of Texas, MD Anderson Cancer Center, Houston, TX, United States

**Keywords:** antibody-dependent cellular cytotoxicity, gynecological cancers, IL-15, monoclonal antibody, natural killer cell, NEO-201, O-glycans

## Abstract

**Background:**

Resistance of gynecological cancers to immunotherapy is due to their ability to impair the cytotoxic activity of immune cells. One strategy to overcome this resistance is the combination of different types of immunotherapies with different mechanisms of action and different targets. The disruption of O-glycosylation pathway in ovarian and endometrial cancer is associated with cancer growth, metastasis, and poor prognosis.

**Methods:**

In this study we treated *in vitro* endometrial and ovarian human cancer cell lines with the combination of the monoclonal antibody (mAb) NEO-201 and IL-15 to overcome the resistance of gynecological cancers to immunotherapy. The combination was also used *in vivo* to treat mice bearing human ovarian cancer. NEO-201 is a humanized IgG1 mAb that binds to core 1 and/or extended core 1 O-glycans expressed by human cancer cells (including different ovarian cancer subtypes), as well as non-cancerous CD15^+^ granulocytes and immunosuppressive cells. NEO-201 can mediate the killing of its target cells through different mechanisms of action, including antibody-dependent cell-mediated cytotoxicity (ADCC) and complement-dependent cytotoxicity (CDC). One strategy to enhance ADCC mediated by mAbs is to boost natural killer (NK) cells with IL-15. A previous study showed that IL-15 superagonist complex (N-803) enhanced ADCC activity mediated by NEO-201 *in vitro* against several human carcinoma cells, by modulating NK cells activation and cytotoxicity.

**Results:**

In this study we demonstrated that IL-15 enhanced ADCC mediated by NEO-201 *in vitro* against human endometrial and ovarian cancer cell lines expressing NEO-201 target antigen, and that the combination of IL-15 and NEO-201, using purified human NK cells as effectors, had a modest effect in prolonging the survival of mice bearing human ovarian cancer, compared to IL-15 or NEO-201 alone.

**Conclusions:**

The ability of IL-15 to enhance NEO-201 efficacy, with NK cells as effectors, supports the hypothesis of combining NEO-201 and IL-15 with NK cell therapy (i.e. IL-15-secreting CAR-NK cells with a longer IL-15 *in vivo* half-life and stronger NK activity) for the treatment of gynecological cancers expressing O-glycans recognized by NEO-201.

## 1 Introduction

Gynecologic cancers include malignancies affecting the ovaries, uterus, cervix, vulva, and vagina (1). The global morbidity and mortality of these cancers still represent a challenge in oncology, with hundreds of thousands of new cases and deaths reported annually (2). Despite the availability of several types of treatment, including surgery, chemotherapy, targeted therapy and immunotherapy, many of these cancers show resistance to the treatment, with progression or relapse of the disease (3).

While immune checkpoint inhibitors (ICIs) have shown modest success as single agents in clinical trials, cancer cells often develop resistance, limiting their effectiveness (3–6). The combination of different types of immunotherapies, such as monoclonal antibodies (mAbs), cytokines, cancer vaccines, adoptive cell therapies and bispecific antibodies, could be a promising strategy to enhance the efficacy of immunotherapy and to overcome the resistance to ICIs in patients with gynecological cancers (4).

One promising strategy to overcome the resistance to immunotherapy in patients with gynecological cancers can be the combination of immunotherapies with different mechanisms of action and different targets (4). In this regard, in recent years it has been demonstrated that one of the pathways disrupted in ovarian and endometrial cancer is the O-glycosylation pathway (7, 8). The O-glycosylation pathway is a post-translational modification which occurs with the addition of a single N-acetyl galactosamine (GalNAc) to serine or threonine residue of mammalian proteins (9). Dysregulation of the O-glycosylation pathway allows cancer cells to express truncated O-glycans and is associated with malignant transformation, cancer growth and the ability of cancer cells, especially those from epithelial cancers, to metastasize (10, 11). The aberrant expression of truncated O-glycans by cancer cells is strongly correlated with a poor prognosis in several types of cancers, including gynecological cancers (12–16). For this reason, the development of new immunotherapeutics targeting O-glycans is becoming a novel and promising strategy to improve the efficacy of immunotherapy in solid tumors, including gynecological cancers (17, 18).

In this context, a logical approach can be the enhancement of the mAb NEO-201’s primary ADCC mechanism by stimulating NK cell activation with IL-15.

NEO-201 is a humanized IgG1 mAb derived from the Hollinshead allogeneic colorectal cancer vaccine platform containing biologically active and immunogenic tumor associated antigens (TAAs) isolated from tumor membrane fractions extracted from several colon cancer tissues. The Hollinshead cancer vaccine was used to generate mAbs in mice. One of the mAbs with the strongest binding and activity against targets expressing TAAs contained in the vaccine was NEO-201 (19). Previous studies have shown that NEO-201 reacts to several human cancer cell lines and tumor tissues, including colon, pancreas, adenocarcinoma of the lung, squamous cell lung cancer, and breast cancer (20, 21). Regarding gynecological cancers, NEO-201 has been found reactive to different ovarian cancer subtypes, with mucinous adenocarcinoma showing the highest percentage of positive samples among all the histological subtypes analyzed (68.2%) (22). Interestingly, NEO-201 did not show reactivity to human healthy epithelial tissues or human healthy tissues surrounding the tumor in immunohistochemistry (IHC), suggesting that NEO-201 binds specifically only to human tumor tissues in a solid tumor environment (19–22).

Preclinical studies demonstrated that NEO-201 binds to core 1 and/or extended core 1 O-glycans expressed by human cancer cells, as well as non-cancerous CD15^+^ granulocytes and immunosuppressive cells, such as regulatory T cells (Tregs) and granulocytic myeloid-derived suppressor cells (gMDSCs) (19, 23).

NEO-201 can mediate the killing of its target cells through different mechanisms of action, including antibody-dependent cell-mediated cytotoxicity (ADCC), complement-dependent cytotoxicity (CDC) and blockade of the interaction between CEACAM5 on tumor cells and CEACAM1 on NK cells (19). NEO-201 showed ADCC activity against cancer cells expressing core 1 and extended core 1 O-glycans and against gMDSCs (19).

One strategy to enhance ADCC mediated by mAbs is to boost NK cells with IL-15. IL-15 stimulates NK cell development, proliferation, cytotoxicity, and cytokine production, including gamma interferon, granulocyte/macrophage colony-stimulating factor (GM-CSF), and tumor necrosis factor alpha (TNF-α) (24). IL-15 also increases the ability of mAbs to mediate ADCC using NK cells as effectors. For example, combination therapy of IL-15 with rituximab in a syngeneic mouse model of lymphoma transfected with human CD20 and with alemtuzumab (Campath-1H) in a xenograft model of human adult T cell leukemia (ATL) showed that IL-15 enhanced the therapeutic efficacy of both rituximab and alemtuzumab in mice (25). These results were confirmed in clinical trials (26). A previous study also showed that IL-15 superagonist complex (N-803) enhanced ADCC activity mediated by NEO-201 *in vitro* against several human carcinoma cells, by modulating NK cells activation and cytotoxicity, suggesting a possible clinical use of IL-15 superagonists in combination with NEO-201 for the treatment of human carcinomas (27).

This study aims to assess the ability of IL-15 to enhance the ADCC activity of NEO-201 against gynecological cancers expressing NEO-201 target antigen and to evaluate if the combination of IL-15 and NEO-201, using purified human NK cells as effectors, can prolong the survival of mice bearing human ovarian cancer compared to IL-15 or NEO-201 alone.

## 2 Materials and methods

### 2.1 Drugs

NEO-201 was generated and provided by Precision Biologics, Inc., Bethesda, MD, USA (19, 20). Recombinant human IL-15 used in this study was provided by National Institutes of Health (NIH)-Cancer Therapy Evaluation Program (CTEP) as a sterile filtered solution endotoxin-free in aqueous buffer.

### 2.2 Cell lines and cultures

The following human cancer cell lines were obtained from the American Type Culture Collection (ATCC): CFPAC-1 (pancreatic); OV-90 (ovarian); HCT116 (colon); KLE CRL-1622 (endometrial).

The following human cancer cell lines were obtained from the National Cancer Institute (NCI): ACI52, ACI80, ACI98, ACI126, ACI158, ARK-1, ARK-2, EC1, EC2, EFE184 (endometrial); TC4601 (non-transformed endometrial epithelium); OVCAR-8 (ovarian).

All human carcinoma cell lines were grown and maintained in a culture medium (Corning Life Science, Manassas, VA, USA) designated by the provider supplemented with 10% USA-sourced and heat-inactivated HyClone Fetal Bovine Serum (FBS; GE Healthcare Life Sciences, Issaquah, WA, USA), 1% penicillin/streptomycin (Corning Life Science, Manassas, VA, USA) and maintained at 37 °C in incubator under 5% CO_2_.

Human cancer cells used for tumor induction in mice were tested by Molecular Testing of Biological Materials (MTBM) as required by the NCI ACUC Committee and confirmed to contain no mouse viruses.

### 2.3 Flow cytometry

Expression of tumor antigen recognized by NEO-201 on human cancer cell lines was analyzed by flow cytometry. Human cancer cell lines (1.0 × 10^6^) were harvested and first incubated with 1 μL per test of LIVE/DEAD Fixable Aqua (Thermo Fisher Scientific, Waltham, MA, USA) in 1× phosphate-buffered saline (PBS) for 30 min at 4°C to accomplish live vs. dead cell discrimination. Cells were then centrifuged, washed twice with cold PBS, and then stained in 1× PBS + 1% BSA (Teknova, Hollister, CA, USA) for 30 min at 4°C with Pacific Blue-conjugated NEO-201 antibody (BioLegend, San Diego, CA, USA).

After staining, cells were washed twice with cold PBS and examined using BD FACSCanto II flow cytometer (BD Biosciences, San Jose, CA, USA). Analysis of cellular fluorescence was performed using BD FACSDiva software (BD Biosciences, San Jose, CA, USA). Percentage (%) of NEO-201 positive cells was calculated with the following formula: % NEO-201 stained cells (cell reactive with NEO-201 in the stained sample) - % unstained cells (% of cells with autofluorescence in the unstained sample). Cells with staining values >10% were considered positive for NEO-201 reactivity. Relative median fluorescence intensity (MFI) was calculated with the following formula: MFI NEO-201 stained cells/MFI unstained cells.

### 2.4 Immunohistochemistry

Formalin-fixed, paraffin-embedded (FFPE) sections of human tumor tissues and human endometrial cancer cell lines were analyzed for NEO-201 target antigen expression using an immunohistochemistry protocol previously described (22).

Slides were digitalized at 20× objective (0.5 × 0.5µm per pixel) using Aperio AT2 scanner (Leica Biosystems) and analyzed using HALO (Indica Labs, v3.6). The appropriateness of staining and regions of interest annotation were completed by a board-certified pathologist to include viable tumor and exclude tumor necrosis, non-tumor tissues, and histology artifact. Quantification of tumor positivity and immune cell infiltrates were performed using image analysis, which included identifying stain vectors, optimizing cell detection algorithms, and thresholding chromogenic IHC staining based on positive and negative controls.

Tissues and cancer cell lines were scored for the expression of the antigen recognized by NEO-201 and percentage of positive tumor cells.

For quantification of tumor expression of NEO-201 target antigen, tissues and cancer cell lines were binned based on staining intensity as negative, 1+ (mild staining; tumor tissues or cancer cell lines with a complete staining of the membrane in <10% of the sample analyzed), 2+ (moderate staining), 3+ (strong staining). Tissues and cancer cell lines with 2+ and 3+ staining intensity were defined as tumor tissues or cancer cell lines with a complete staining of the membrane in more than 10% of the sample analyzed.

The % of positive cells was determined for each bin and the tumor H-score was calculated as follow: H-score = [1*(%1+) + 2*(%2+) + 3*(%3+)] (28).

Tissue microarrays (TMA) of 185 endometrial tumor samples were obtained from Dr. Faceuglia P. at University of Southern California.

TMA were immunolabeled with NEO-201, CD4, and FoxP3 monoclonal antibodies using multiplex immunofluorescence. DAPI was used for fluorescent nuclear staining and Gold antifade mounting media was used with coverslips. Whole slide fluorescent imaging was performed with a Akoya Phenoimager HT scanner with a 20x objective (0.5 × 0.5µm per pixel) and analyzed using QuPath (v 0.5.1) (29). Digital image analysis included identifying stain vectors, de-arraying tissue microarrays, defining regions of interest, detecting cells, excluding artifact and normal adjacent tissues, and quantifying immunofluorescent signal. Following automated TMA grid detection, manual pathologist review was performed to exclude spots or regions of spots that were non-assessable, non-tumor containing, or obscured by artifact. Nuclei segmentation and cell detection was performed using a pretrained convolutional neural network model employing a star-convex polygon approach. Staining was quantified for detected cells and thresholds were determined for each assay to bin cells based on fluorescent intensity as follows: 0 for no staining, 1 for mild staining, 2 for moderate, and 3 for strong staining. Tumor H-scores were calculated as follows: H-score = [1*(%1+) + 2*(%2+) + 3*(%3+)] (28).

### 2.5 Exome sequencing of human endometrial cancer cell lines

Exome samples were pooled and sequenced on HiSeq using Agilent SureSelect Human All Exon V7 and paired-end sequencing. The samples had 150M to 194M pass filter reads, with Q30 above 93%. The samples were mapped, and variants were called using DRAGEN (Dynamic Read Analysis for GENomics) pipeline provided by the CCR sequencing facility at NCI (https://crtp.ccr.cancer.gov/sf/). Percent total mapping against reference genome Human - hg38 is about 99% and Uniquely mapped reads were above 70%. Library complexity (i.e. percentage of non-duplicate reads) was determined by measuring the percentage of unique fragments in the mapped reads using MarkDuplicate utility. Percent duplicate reads are between 23% to 29%. There are 85% to 87% of reads mapped on target. Coverage statistics were also measured using DRAGEN. Raw sequencing depth coverage over the target region was between 457x and 589x and mapped sequencing depth coverage over target (after alignment and marking duplicates) was between 215x to 276x. The mean insert size for these samples was between 180 and 200 bases. More than 95% of the target region have the coverage above 20x. DRAGEN was run for germline joint genotyping. There are about 40K to 58K germline joint genotyping variants per sample, including 37K to 50K SNPs and 3K to 9K INDELs. The Ti/Tv ratio was between 2.5 and 2.7.

### 2.6 ADCC assay

The ADCC activity of NEO-201 against human carcinoma cell lines was evaluated using a radioactive ADCC assay. The following human cancer cell lines were used as target cells: CFPAC-1 (pancreatic cancer cell line; positive control), OVCAR-8 (ovarian cancer cell line; negative control), OV-90 (ovarian cancer cell line), ACI158 (endometrial cancer cell line).

Human peripheral blood mononuclear cells (PBMCs) and isolated NK cells from the same healthy donor were used as effector cells.

PBMCs were collected from anonymous healthy donors under protocol 99-CC-0168, approved by the Institutional Review Board of the National Cancer Institute.

NK cells were isolated from PBMCs from healthy donors using the EasySep Human NK Cell Isolation Kit (StemCell Technologies, Vancouver, BC, Canada) according to the manufacturer’s protocol.

Before the ADCC assay, both PBMCs and NK cells were treated with recombinant human IL-15 at different concentrations (2, 1, 0.5 and 0.25 ng/mL) or vehicle control medium (RPMI-1640 medium supplemented with l-glutamine, 10% FBS, and 1% penicillin/streptomycin) for 48 hours.

On the day of the assay, target cells (2x10^6^) were pulsed with 60µCi 111In-oxyquinoline (GE Healthcare) at 37°C for 20 minutes and then 3,000 cells per well were plated in 96-well round-bottom culture plates in vehicle control medium.

Then PBMCs were added at effector cell-to-target cell (E:T) ratios of 50:1 and NK cells at E:T ratios of 12:1 and 6:1 in presence of 10 µg/mL of NEO-201 or human IgG1 isotype control antibody (Thermo Fisher Scientific, Waltham, MA, USA).

For blocking studies, on the day of the ADCC assay, purified NK cells untreated or treated with IL-15 (0.25 ng/mL) were incubated at 37°C for 2h with 15 µg/mL of anti-human CD16-neutralizing mAb (eBioscience, San Diego, CA) prior to being used as effectors at 6:1 E:T ratio.

Spontaneous release was determined by incubating target cells with vehicle control medium alone, and complete lysis by incubation with 0.05% Triton X-100.

After 4h incubation at 37°C under 5% CO_2_, cells were harvested and 111In-oxyquinoline release was detected using a specific gamma counter.

Specific ADCC lysis was determined using the following equation:


$$\text{Percent lysis }=\left(\text{experimental sample release }–\text{ spontaneous release}\right)/\left(\text{complete release }-\text{ spontaneous release}\right)\text{ x }100$$

### 2.7 Assessment of IL-15 ability to enhance NEO-201 efficacy on survival *in vivo*

*In vivo* experiments were performed on 6–8 weeks old female athymic nude mice. Mice were maintained on a 12h light/dark cycle, with food and water provided ad libitum.

To assess the IL-15 ability to enhance NEO-201 efficacy on survival, 1 × 10^6^ OV-90 cells were injected into the peritoneal cavity of each mouse. Tumors were allowed to grow for 2 weeks before 9 mice per group were randomized into the following groups:

Group 1: NK cells + NEO-201 (9X10^5^ NK cells/mouse + 250μg/mouse NEO-201)
Group 2: NK cells + NEO-201 + IL-15 (9X10^5^ NK cells/mouse + 250μg/mouse NEO-201 + 5μg/mouse IL-15)
Group 3: NK cells + IL-15 (9X10^5^ NK cells/mouse + 5μg/mouse IL-15)

NEO-201 was administered IP on days 1, 4, and 8 of treatment, while NK cells were administered IP on days 2, 5, and 9 together with IL-15. Before injection, isolated NK cells were cultured overnight in vehicle control medium. After the first treatment, mice were followed for survival for 120 days. Mice were evaluated biweekly for signs of drug-related toxicity and disease progression based on distress, physical exam changes, and cachexia. Animal care was provided in accordance with the procedures in the Guide for the Care and Use of Laboratory Animals. Experiments were carried out according to a protocol approved by the National Cancer Institute Animal Care and Use Committee.

### 2.8 Statistical analysis

Differences between multiple treatments in the *in vitro* ADCC assay were evaluated by 2way ANOVA followed by Turkey’s multiple comparison test, using GraphPad Prism 10.0.2 software (GraphPad Software, La Jolla, CA, USA). Differences were considered significant when the p value was < 0.05.

Survival curves were analyzed using the Kaplan–Meier method and compared using the log-rank (Mantel-Cox) test using GraphPad Prism 10.0.2 software. Differences in survival curves were considered significant when the p value was < 0.05.

## 3 Results

### 3.1 Expression profile of NEO-201 binding in human endometrial and ovarian cancer cell lines by flow cytometry

Flow cytometry analysis was used to profile a panel of human endometrial and ovarian cancer cell lines for NEO-201 binding. The staining profile is summarized in **Table 1**.

**Table 1:** Flow cytometry analysis of NEO-201 binding to cultured cancer cell lines derived from human endometrial and ovarian cancers.

Cell line	Tumor type	% unstained (MFI)	% NEO-201 stained (MFI)	% NEO-201 positive	Relative MFI
ACI158	**Endometrial serous adenocarcinoma**	2.6 (402)	74.2 (1,150)	**71.6**	2.86
ACI80	**Endometrial adenocarcinoma**	4.7 (300)	30.3 (265)	**25.6**	0.88
ACI52	Endometrial adenocarcinoma	4.7 (498)	7.0 (505)	2.3	1.01
ACI98	Endometrial undifferentiated carcinoma	4.0 (479)	9.4 (481)	5.4	1.00
ACI126	Endometrial serous adenocarcinoma	5.5 (481)	9.5 (482)	4.0	1.00
ARK-1	Endometrial serous adenocarcinoma	1.7 (476)	8.2 (501)	6.5	1.05
ARK-2	Endometrial serous adenocarcinoma	1.4 (911)	4.4 (972)	3.0	1.07
EC1	Endometrial adenocarcinoma	5.2 (697)	9.7 (727)	4.5	1.04
EC2	Endometrial clear cell carcinoma	8.0 (1,445)	9.5 (1,498)	1.5	1.04
OV-90	**Ovarian papillary serous adenocarcinoma**	2.0 (495)	**32.4** (647)	**30.4**	1.31
OVCAR-8	Ovarian high grade serous adenocarcinoma	0.2 (187)	8.4 (79)	8.2	0.42
CFPAC-1	**Pancreatic ductal adenocarcinoma** **(positive control)**	1.2 (335)	99.1 (5,931)	**97.9**	17.70

The percentage of unstained, NEO-201 stained, NEO-201positive cells, median fluorescence intensity (MFI) and relative MFI values are detailed for each cell line. Percentage (%) of NEO-201 positive cells was calculated with the following formula: % NEO-201 stained cells (cell reactive with NEO-201 in the stained sample) - % unstained cells (% of cells with autofluorescence in the unstained sample). NEO-201 positivity was defined as % positive >10%. NEO-201 positive cell lines appear in bold text.

Relative MFI was calculated with the following formula: MFI NEO-201 stained cells/MFI unstained cells.

The human pancreatic cancer cell line CFPAC-1 was used as positive control for NEO-201 binding, since previous studies showed that this cell line is highly positive for NEO-201 binding in flow cytometry (20, 27).

Assessment of the binding activity of NEO-201 revealed that only 2/9 (22%) of human endometrial cancer cell lines (ACI80 and ACI158) were positive for NEO-201 binding. The cell line with the strongest reactivity for NEO-201 was ACI158 (71.6% of cells positive) (**Table 1**).

Between the two ovarian cancer cell lines tested, OV-90 was found to be reactive with NEO-201 (30.4%), while the cell line OVCAR-8 was negative for NEO-201 binding (**Table 1**). Representative flow cytometry plot for each cell line is shown in **Supplementary Figure 1**.

### 3.2 Expression profile of the antigen recognized by NEO-201 in human endometrial tumor tissues by IHC

**Figure 1:**
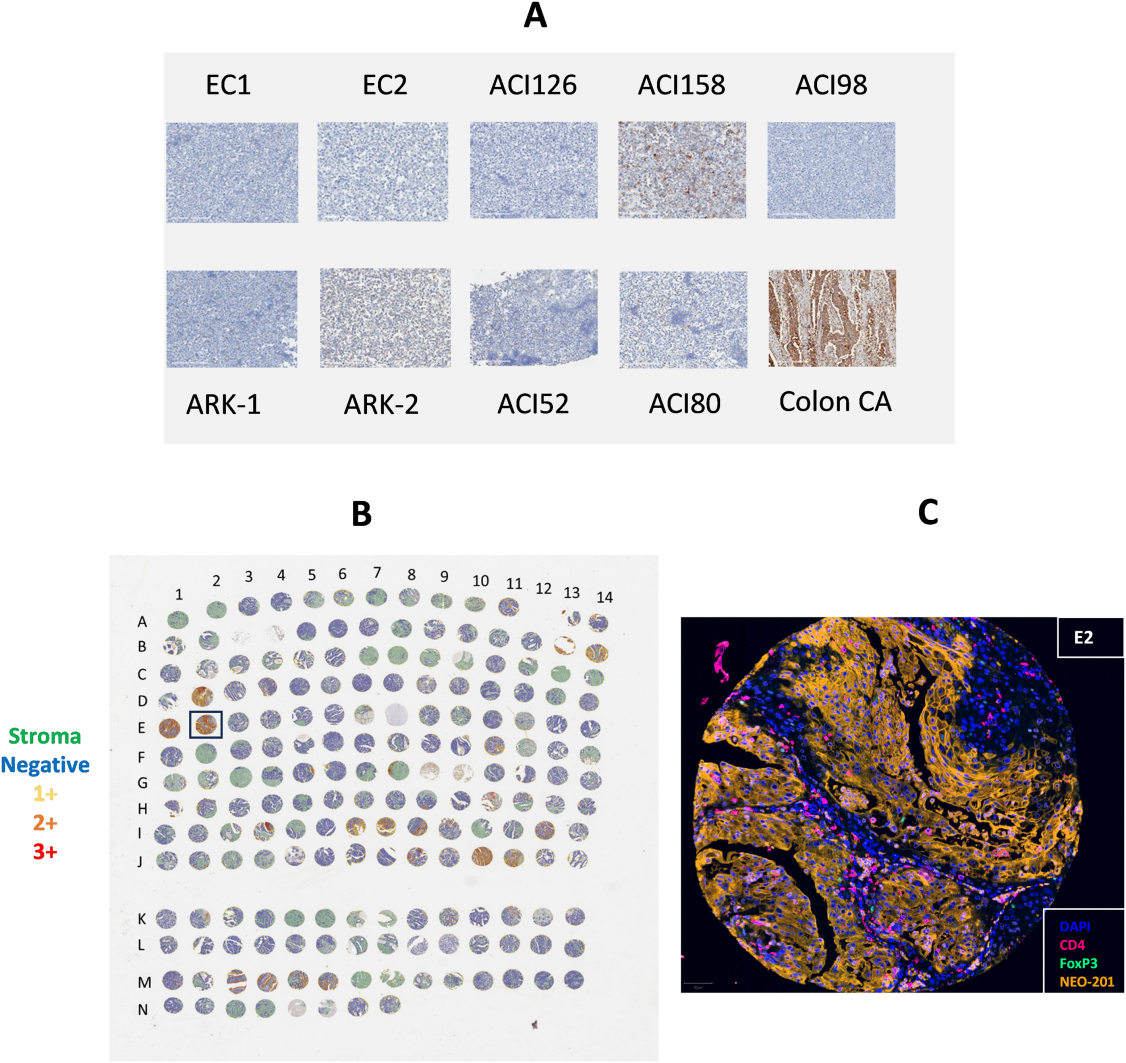
Expression profile of the antigen recognized by NEO-201 in human endometrial tumor tissues by IHC. NEO-201 binding was evaluated by IHC in several human endometrial cancer cell lines. **(A)** Representative NEO-201 staining of human endometrial cancer cell lines with a colon cancer tissue used as positive control. For quantification of tumor expression of NEO-201 target antigen, tissues and cancer cell lines were binned based on staining intensity as negative, 1+ (mild staining; tumor tissues or cancer cell lines with a complete staining of the membrane in <10% of the sample analyzed), 2+ (moderate staining), 3+ (strong staining). Tissues and cancer cell lines with 2+ and 3+ staining intensity were defined as tumor tissues or cancer cell lines with a complete staining of the membrane in more than 10% of the sample analyzed. All images were obtained at 20X **(B)** Tissue microarrays (TMA) of 185 endometrial tumor samples. TMA were immunolabeled with NEO-201, CD4, and FOXP3 monoclonal antibodies using multiplex immunofluorescence. Staining was quantified for detected cells and thresholds were determined for each assay to bin cells based on fluorescent intensity as follows: 0 for no staining, 1 for mild staining, 2 for moderate, and 3 for strong staining. Tumor H-scores were calculated as follows: H-score = [1*(%1+) + 2*(%2+) + 3*(%3+)]. **(C)** Enlargement of a human endometrial tumor sample in the TMA reactive with NEO-201, CD4 and FoxP3 monoclonal antibodies.

NEO-201 binding profile on ovarian cancer was already evaluated by TMA in a previous manuscript, where we reported the reactivity of NEO-201 in 627 tissues from more than 10 different ovarian cancer subtypes including serous, germ cell, clear cell, endometroid, mucinous, sarcoma, transitional, and adenocarcinoma (22). In this manuscript, to confirm the data obtained in flow cytometry, NEO-201 binding was evaluated by IHC in several human endometrial cancer cell lines. A colon cancer tissue was used as positive control (**Figure 1A**). IHC confirmed results from flow cytometry.

Among all human endometrial cancer cell lines analyzed, only the cell line ACI158 (endometrial serous adenocarcinoma) reacted with NEO-201 in IHC (18.30% cells positive for NEO-201 staining, with H-score of 20.95). The cell line ACI80, that was NEO-201 positive in flow cytometry, did not react with NEO-201 in IHC. All other human endometrial cancer cell lines analyzed by IHC had less than 2% of cells positive for NEO-201 staining (**Supplementary Table 1**).

The binding of NEO-201 was also evaluated in tumor samples, using a TMA containing 185 endometrial tumor samples derived from subjects with uterine cancer. Types of uterine cancers included in the TMA were endometrioid G1 (118) and G2 (67) (**Supplementary Table 2**).

Many of the tissues analyzed derived from patients with FIGO (International Federation of Gynecology and Obstetrics) stage IA and IB (**Supplementary Table 2**).

Analysis of TMA showed that a total of 16 tissues had more than 10% of NEO-201 positive cells. In particular, 10 tissues had 10-24% of cells NEO-201 positive, 2 tissues had 25-49% of NEO-201 positive cells (**Figure 1B**; D4 and M4), 2 tissues had 50-74% of NEO-201 positive cells (**Figure 1B**; B13 and M3), and 2 tissues had 75-100% of NEO-201 positive cells (**Figure 1B**; E1 and E2).

A strong membranous staining by NEO-201 at the level of apical surfaces of glandular structures of endometrial tumor tissues was observed in most of these 16 tissues.

Occasionally, limited staining was observed at the level of apical surfaces of columnar epithelial cells. A positive staining was also observed within glandular lumina. This may indicate the presence of inflammatory cells infiltrating the lumina or potentially sloughed epithelial cells or membrane secretions from the endometrial glandular epithelium (**Figures 1B, C**).

A deeper analysis of endometrial cancer tissue represented in **Figure 1B** (E2) revealed also the presence of cells consistent with infiltrating immune cells in the lumina. Staining of this specific endometrial tumor tissue infiltrated with immune cells showed that NEO-201 stained only the membrane and cytoplasm of tumor cells (79.5% tumor cells positive for NEO-201), while the immune cells were primarily CD4^+^ (**Figure 1C**). A very small fraction of CD4^+^ cells were also FoxP3^+^ (0.02%), indicating a limited infiltration of tumor tissue by regulatory T cells (Tregs). However, CD4^+^ and CD4^+^/FoxP3^+^ cells were negative for NEO-201.

### 3.3 Mutational analysis

To evaluate the correlation between genetic mutations carried by gynecological tumors and their sensitivity or resistance to NEO-201, we performed whole exome and RNA sequencing on different human endometrial cancer cell lines, including cell lines positive or negative for NEO-201 binding in IHC.

We searched for mutations that were commonly present in endometrial cancer cell lines reactive with NEO-201 but not in endometrial cancer cell lines not reactive with NEO-201, or for mutations specifically expressed in a particular cancer cell line.

**Figure 2:**
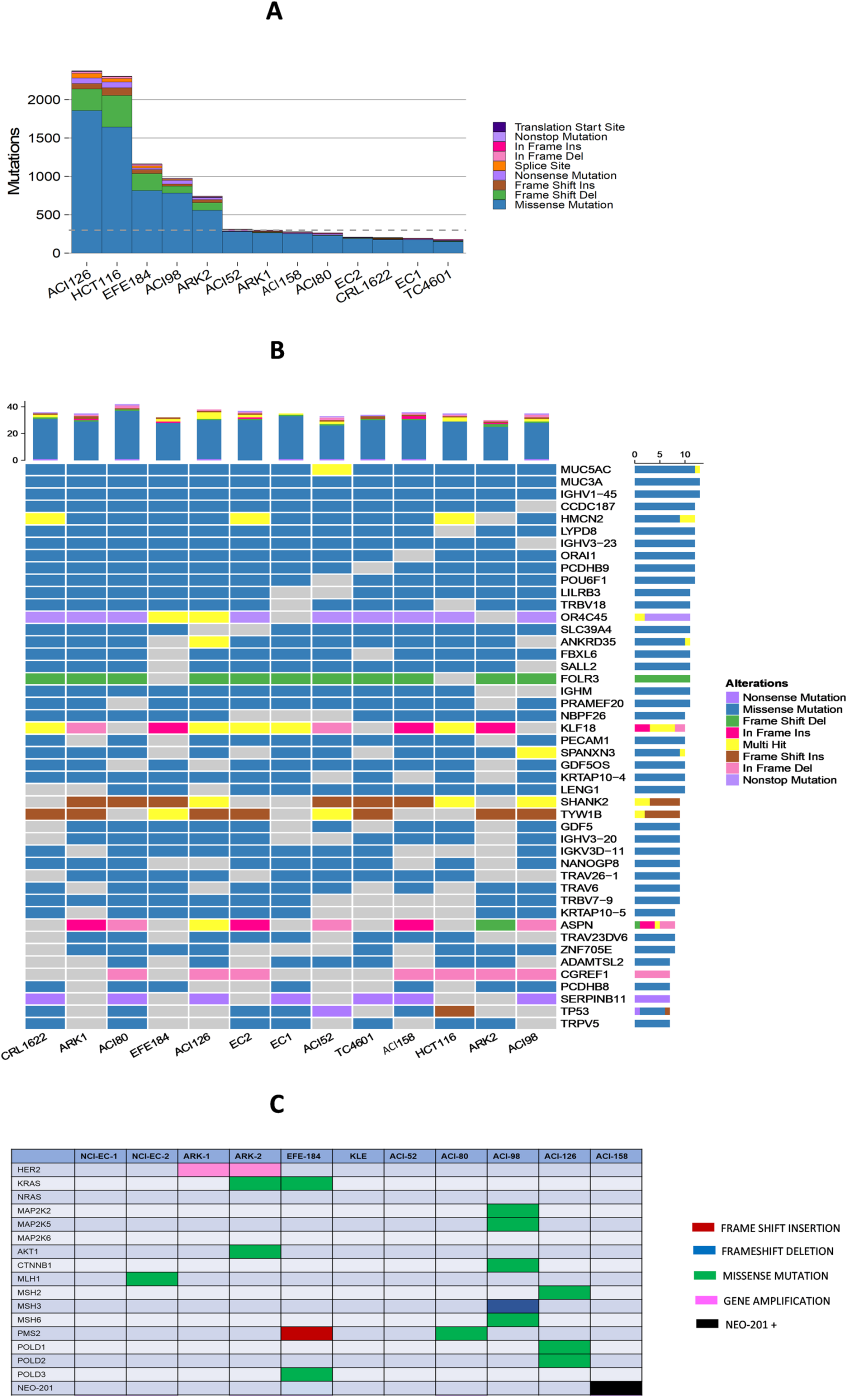
Exome sequencing of human endometrial cancer cell lines. Whole exome and RNA sequencing of human endometrial cancer cell lines positive or negative for NEO-201 binding in IHC. **(A)** Mutation burden with number of mutations detected in each cancer cell line. **(B)** Oncoplot depicting scale and types of genetic alterations found in each cancer cell line. **(C)** Oncoplot depicting types of variants of interest found in each cancer cell line.

The mutation burden varied widely across cell lines, with the highest number of mutations in uterine serous cell line ACI126 and lowest in endometrial adenocarcinoma line EC1 (**Figure 2A**). The human colon cancer cell line HCT116 was used as positive control for the mutational burden since these cells exhibit microsatellite instability (MSI) and an increased rate of mutations. HCT116 showed higher mutational burden than control untransformed human endometrial epithelial cells TC4601 (low mutational count) (**Figure 2A**). Alterations were predominantly made up of missense mutations or frame-shift deletions. Histological subtype of origin (serous or non-serous) did not correlate with mutational burden (**Figure 2B**).

Focusing down on specific mutations commonly found in this dataset, the mucin-related genes MUC5AC and MUC3A universally carried missense mutations in all the cell lines. Of note, the genes ORC4C45 (nonsense mutation) and FOLR3 (frame shift deletion) appeared to be commonly inactivated in these cell lines, suggesting the possibility of tumor-suppressor functions (**Figure 2B**).

Several genes were more closely examined based on their known association with colon and endometrial cancers and for possible combination strategies (**Figure 2C**). We evaluated whether any of these were found in the ACI158 cell line that was highly reactive with NEO-201. Interestingly, we observed that the ACI158 cell line (only cell line reactive with NEO-201 in IHC) and EC1 cell line (not reactive with NEO-201 in IHC) do not carry any missense mutation, gene amplification, frame shift deletion or insertion in this set of genes considered as variants of interest. On the contrary, in all the other endometrial cancer cell lines that do not react with NEO-201 in IHC we observed the following mutations in variants of interest: amplification of HER2 gene in ARK-1 and ARK-2 cells; mutations of Mismatch Repair (MMR) genes (MLH1, MSH2, MSH3, MSH6, PMS2) in EC2, ACI80, ACI98, ACI126 cell lines; mutation of KRAS and AKT1 gene in ARK-2 cell line; mutation of mitogen-activated protein kinase (MAPK) genes (MAP2K2, MAP2K5) and CTNNB1 gene in the ACI98 cell line (**Figure 2C**).

### 3.4 IL-15 enhances ADCC mediated by NEO-201 against human endometrial and ovarian cancer cell lines *in vitro*

The ability of NEO-201 to mediate ADCC against human cancer cell lines expressing its target antigen has been demonstrated in previous studies (20, 21, 27).

In this study we evaluated the ability of NEO-201 to mediate ADCC in gynecological cancers using the following cell lines tested for the expression of NEO-201 target antigen in flow cytometry: CFPAC-1 (97.9% of cells expressing NEO-201 target antigen): positive control; OVCAR-8 (8.2% of cells expressing NEO-201 target antigen): negative control; OV-90 (30.4% cells expressing NEO-201 target antigen) and ACI158 (71.6% cells expressing NEO-201 target antigen).

Before the ADCC assay, both PBMCs and NK cells, isolated from PBMCs from same healthy donors, were treated with recombinant human IL-15 at different concentrations (2, 1, 0.5 and 0.25 ng/mL) for 48 hours. These experiments show that IL-15 enhanced statistically NEO-201-mediated ADCC against CFPAC-1 at 1ng/mL (p<0.01), OV-90 at 2 ng/mL (p<0.01), and ACI158 at 2 and 1ng/mL (p<0.01) with PBMCs as effectors (**Supplementary Figure 2**).

When NK cells were used as effectors, the best enhancement of NEO-201-mediated ADCC was achieved with IL-15 at 0.25 ng/mL in CFPAC-1 and ACI158 cell lines with NK cells at12:1 E:T ratio (**Supplementary Figure 2**). For this reason, we chose the concentration of 0.25ng/mL for future experiments.

To evaluate the ability of IL-15 to enhance ADCC mediated by NEO-201 with both PBMCs and NK cells as effectors in multiple healthy donors, we treated PBMCs and NK cells from three different healthy donors without and with IL-15 at the concentration of 0.25ng/mL for 48 hours before using them in the ADCC assay.

**Figure 3:**
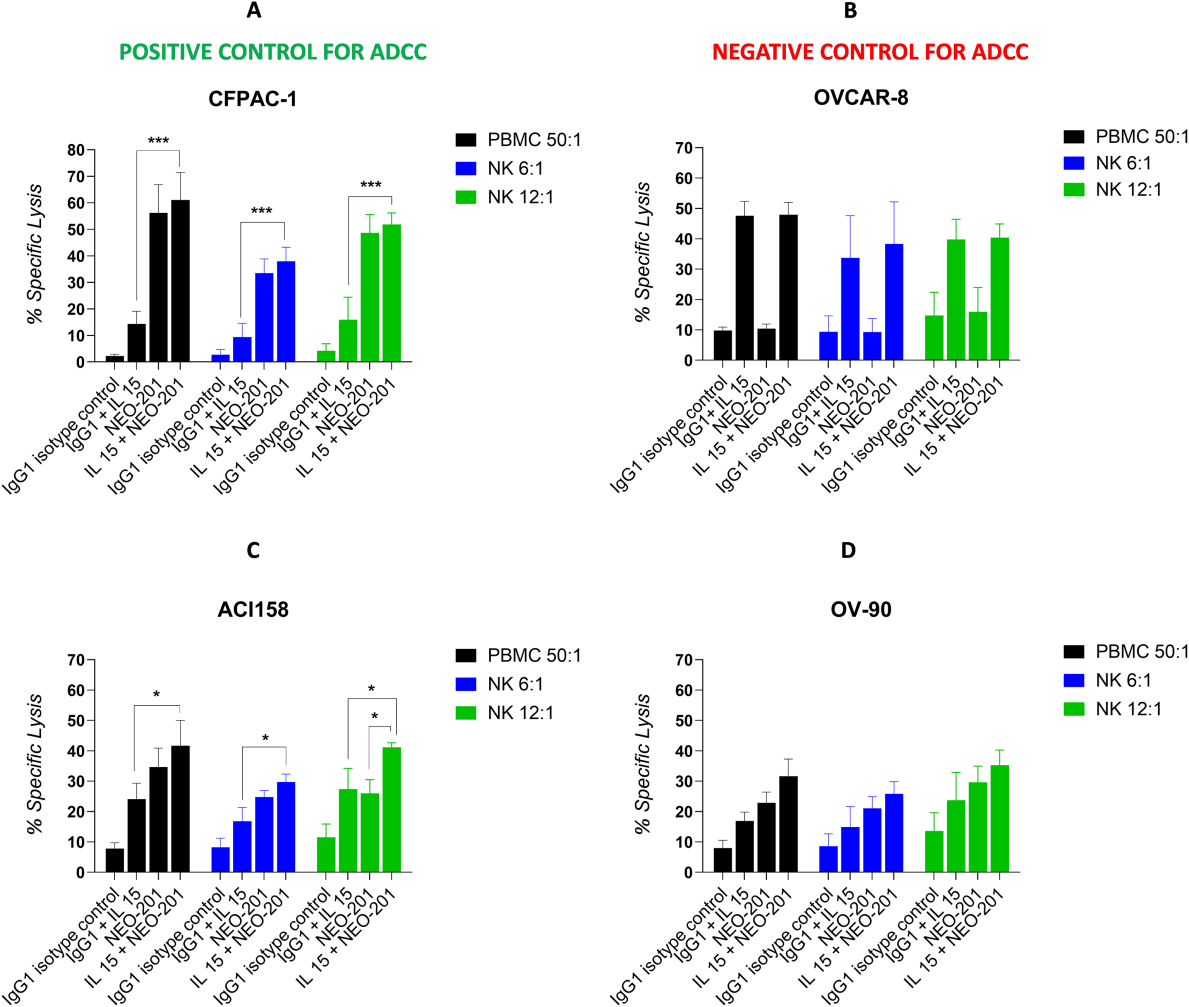
IL-15 enhances the ADCC activity mediated by NEO-201 against CFPAC-1, OV-90, and ACI158 cell lines. Human carcinoma cell lines were used as target cells in the presence of 10 µg/mL of NEO-201 or human IgG1 (isotype control) in the ADCC assay. PBMCs and purified NK cells were treated with IL-15 (0.25 ng/mL) or vehicle control for 48 h before being used as effector cells at the indicated E:T ratios. **(A)** Human pancreatic cancer cell line CFPAC-1. **(B)** Human ovarian high grade serous adenocarcinoma cell line OVCAR-8. **(C)** Human endometrial serous adenocarcinoma cell line ACI158. **(D)** Human ovarian papillary serous adenocarcinoma cell line OV-90. Results are presented as mean ± S.E.M. from three experiments using NK cells and PBMCs from three different healthy donors. For each experiment, the mean of percentage of lysis from two replicate wells achieved by PBMCs and NK cells from one healthy donor (n=1) was calculated. Then the mean ± S.E.M. from three experiments [(n=1 + n=1+ n=1)/3] was calculated and reported in the bar graph for each condition tested. Asterisks denote statistical significance (two-way ANOVA). *p < 0.05; ***p < 0.001.

Results from experiments in **Figure 3** (mean of 3 experiments performed with three different healthy donors) show that NEO-201 mediates the killing of CFPAC-1, OV-90 and ACI158 cancer cell lines via ADCC with both PBMCs and NK cells as effectors. Results also confirmed that OVCAR-8 is an optimal negative control to test the specificity of the ADCC mediated by NEO-201. No difference in tumor lysis between NEO-201 and IgG isotype control was observed with both PBMCs and NK cells as effector cells with or without treatment with IL-15. This shows that NEO-201 does not mediate ADCC against cancer cells that do not express its target antigen (**Figure 3B**).

In cancer cell lines CFPAC-1 and ACI158 the combination of IL-15 + NEO-201 resulted in a statistically significant higher lysis compared to IL-15 alone with both PBMCs and NK cells as effectors (**Figures 3A, C**).

In the cell line ACI158, the mean of percentage of specific lysis from three different healthy donors showed that IL-15 enhanced ADCC mediated by NEO-201 in a statistically significant manner only when NK cells were used as effector cells at 12:1 E:T ratio (**Figure 3C**).

On the other hand, although in both CPFAC-1 and OV-90 cell lines we observed that IL-15 enhanced ADCC mediated by NEO-201 with NK at 12:1 ratio in a statistically significant manner in one healthy donor, this enhancement was not statistically significant when we calculated the mean of results from 3 different healthy donors (**Figures 3A, D**). This phenomenon is due to the variability in percentage of lysis between different donors.

Altogether, these data suggest that IL-15 can enhance ADCC mediated by NEO-201 against gynecological cancers expressing NEO-201 target antigen. Furthermore, since the percentage of lysis via ADCC mediated by NK cells is similar to that mediated by PBMCs in CFPAC-1, OV-90 and ACI-158 cancer cell lines, we decided to use NK cells as effector cells in the *in vivo* experiments.

This observation confirms results of a previous study, which demonstrated that NEO-201 utilizes NK cells as one of the main effectors to mediate ADCC (27).

### 3.5 ADCC mediated by NEO-201 enhanced by treatment of NK cells with IL-15 is dependent on CD16 engagement

In a previous study, we demonstrated that treating NK cells with an anti-CD16 antibody, prior to using them in the ADCC assay, significantly decreased ADCC activity mediated by NEO-201 against the CFPAC-1 cell line when NK cells alone or NK cells boosted with the IL-15 superagonist were used as effector cells (27).

**Figure 4:**
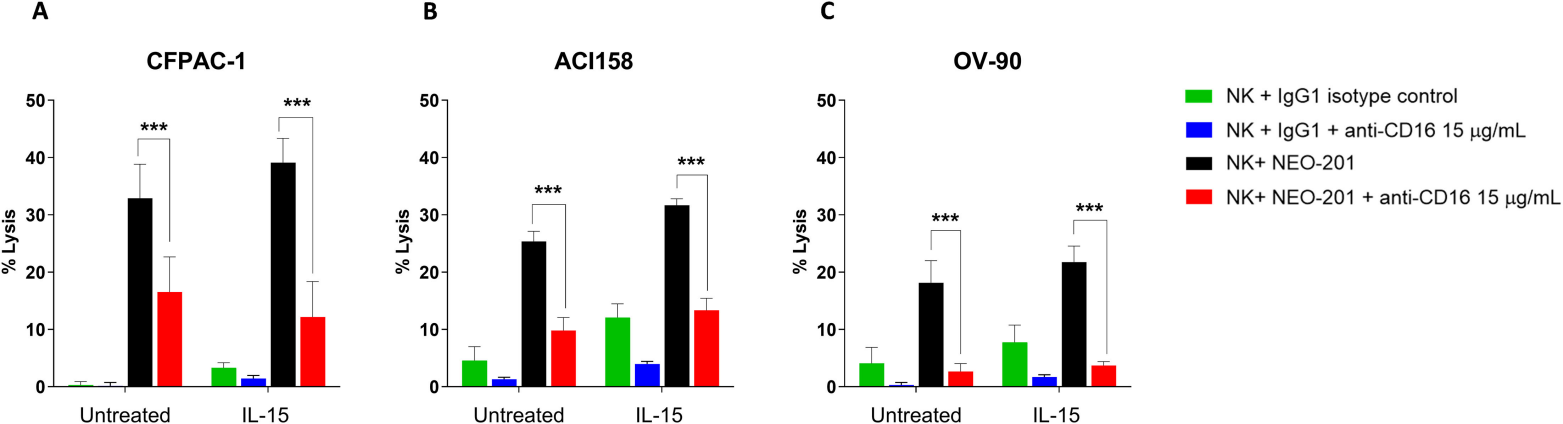
ADCC mediated by NEO-201 and enhanced by IL-15 can be blocked by the anti-CD16 antibody. CFPAC-1, ACI158 and OV-90 cancer cell lines were used as target cells in the presence of 10 µg/mL of NEO-201 or human IgGl (isotype control) in the ADCC assay. Purified NK cells from three healthy donors were treated with IL-15 (0.25 ng/mL) or vehicle control for 48 h before being used as effector cells at the E:T ratio of 6:1. The day of the ADCC assay, some NK cells were pretreated for 2h with anti-CD16 blocking antibody (15 µg/mL) before being used as effectors. **(A)** Human pancreatic cancer cell line CFPAC-1. **(B)** Human endometrial serous adenocarcinoma cell line ACI158. **(C)** Human ovarian papillary serous adenocarcinoma cell line OV-90. Results are presented as mean ± S.E.M. from three experiments using NK cells from three different healthy donors. For each experiment, the mean of percentage of lysis from two replicate wells achieved by NK cells from one healthy donor (n=1) was calculated. Then the mean ± S.E.M. from three experiments [(n=1 + n=1+ n=1)/3] was calculated and reported in the bar graph for each condition tested. Asterisks denote statistical significance (two-way ANOVA). ***p < 0.001.

In this study, to confirm that tumor cells lysis of endometrial (ACI158) and ovarian (OV-90) cancer cell lines is mediated by NEO-201 via ADCC and that IL-15 is able to enhance NEO-201-mediated ADCC, we used NK cells isolated from three healthy donors at 6:1 E:T ratio as effectors cells. NK cells were untreated or treated with IL-15 (0.25 ng/mL) for 48h prior to being used in the ADCC assays. The day of the ADCC assay, the anti-CD16 antibody was added to NK cells 2 hours prior to using them as effector cells. As shown in **Figure 4**, the anti-CD16 antibody significantly decreased ADCC mediated by NEO-201 in both untreated and IL-15-treated NK cells (p<0.001) in all cell lines, confirming that NEO-201 mediates ADCC to kill tumor cells expressing its target antigen and that IL-15 enhances specifically NEO-201-mediated ADCC.

### 3.6 *In vivo* efficacy of NEO-201 + IL-15 in mice bearing human ovarian cancer

To assess the ability of IL-15 to enhance NEO-201 efficacy on overall survival in mice, we used a murine model previously tested for survival *in vivo* studies (22). Briefly, cancer cells from the OV-90 cancer cell line were inoculated into the peritoneal cavity of 6–8 weeks old female athymic nude mice to reproduce the condition of disseminated ovarian cancer and peritoneal carcinomatosis. Tumor-bearing mice were treated with NK cells + NEO-201 with or without IL-15.To have the best efficacy as effector for the ADCC mediated by NEO-201 *in vivo*, we used isolated NK cells from a healthy donor with a good ADCC *in vitro* against OV-90 cells (**Supplementary Figure 3**).

**Figure 5:**
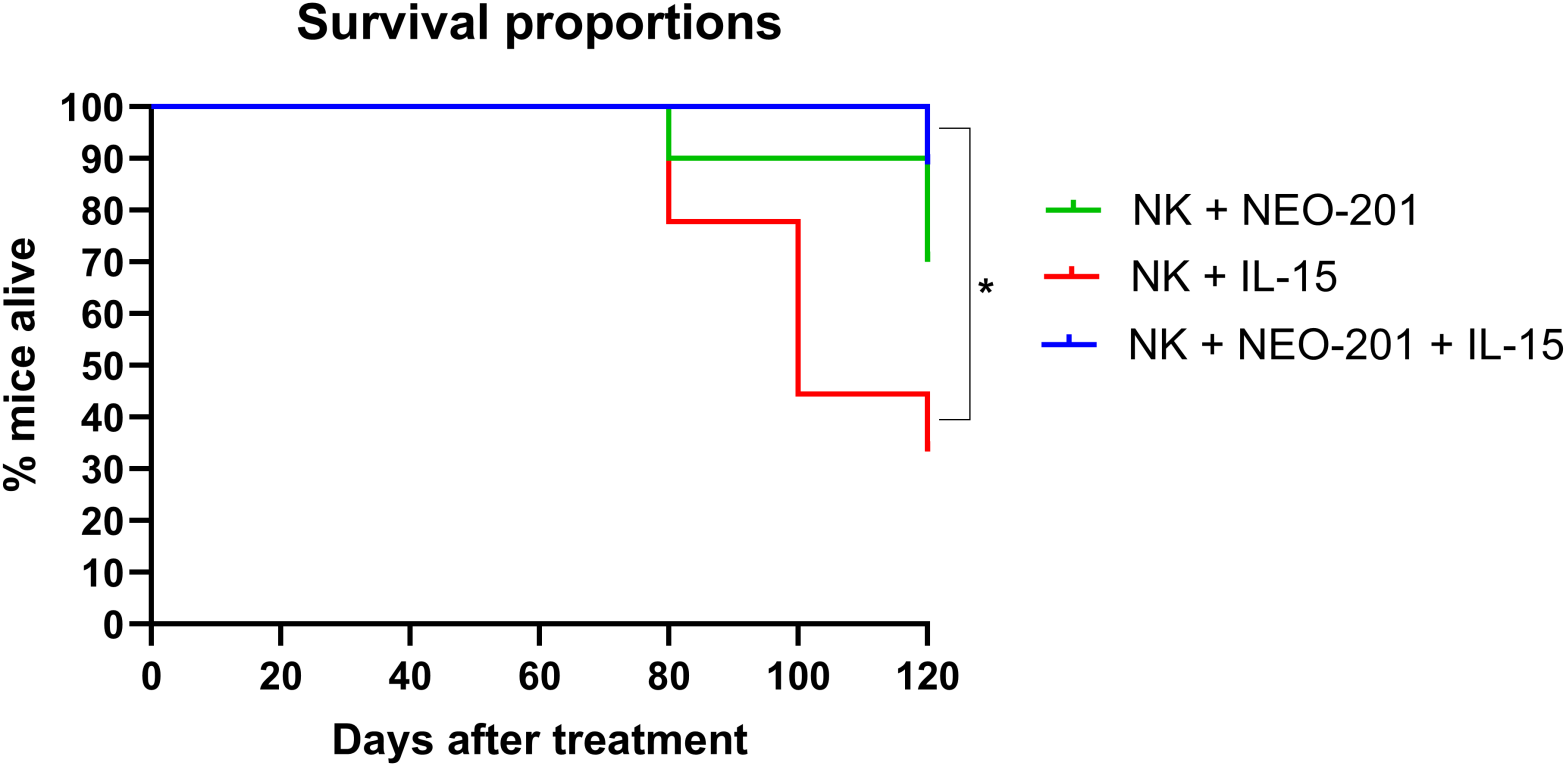
Effect of IL-15 and NEO-201 alone or in combination on survival in murine model of human ovarian cancer *in vivo.* 1×10^6^ OV-90 cells were injected into the peritoneal cavity of each mouse (6–8 weeks old female athymic nude mice). Tumors were allowed to grow for 2 weeks before 9 mice per group were randomized into three different groups of treatment. After randomization, each mice received NEO-201 IP on days 1, 4, and 8 of treatment, NK cells IP on days 2, 5, and 9 together with IL-15. Before injection, isolated NK cells were cultured overnight in vehicle control medium. Isolated NK cells were 10% of PBMCs. After the first treatment, mice were followed for survival for 120 days. Curves represent survival. Survival curves were analyzed using the Kaplan–Meier method and compared using the log-rank (Mantel-Cox) test. Differences in survival curves were considered significant when the p value was < 0.05. *p < 0.05.

After treatments described in materials and methods, tumor-bearing mice were followed for survival for 120 days. Mice treated with NK cells + NEO-201 + IL-15 experienced the longest median survival compared to all other groups (**Figure 5**, **Supplementary Table 3**). Although the risk of dying from cancer in mice treated with NK cells + NEO-201 + IL-15 was 2.92 times lower compared to mice treated with NK cells + NEO-201, the difference in median survival between these two groups was not statistically significant (**Figure 5**, **Supplementary Table 3**).

Instead, mice treated with NK cells + NEO-201 + IL-15 showed a statistically significant prolonged median survival compared to mice treated with NK cells + IL-15 (p=0.0122; 8.34 times lower risk of developing tumors) (**Figure 5**, **Supplementary Table 3**).

It is also important to note that mice treated with NK cells + NEO-201 did not show a statistically significant prolonged median survival compared to mice treated with NK cells + IL-15 (**Figure 5**, **Supplementary Table 3**).

## 4 Discussion

Immunotherapy has become a treatment option that increases the efficacy of cancer therapies across multiple types of cancer, including gynecological cancers. The limitation of immunotherapy in gynecological cancers, especially when immunotherapeutics are administered as single agents, is due to the ability of gynecological cancers to impair the cytotoxic activity of immune cells. Additionally, most gynecologic cancers lack specific genetic profiles, such as deficiency of mismatch repair (MMR) and/or high tumor mutation burden where immunotherapy exerts greater efficacy (1–6). For example, in ovarian cancer, the resistance to immunotherapy is due to limited presence of tumor-infiltrating lymphocytes (TILs), immunosuppressive tumor microenvironment (TME), low percentage of patients with high microsatellite instability and high tumor mutation burden (5).

Major histocompatibility complex (MHC) class I loss is a potential mechanism of resistance to immune checkpoint inhibition in endometrial carcinoma. MHC class I loss by endometrial carcinoma cells reduces the presentation of tumor neoantigen to cytotoxic T cells and can result in a mechanism of resistance to checkpoint inhibitors even in mismatch repair (MMR)-deficient and PD-L1-positive endometrial cancers (6). Glycosylation is one of the most complex post-translational modifications of mammalian cells and is essential in cellular processes such as cell-matrix interaction, cell-to-cell recognition and intracellular signaling. O-glycosylation is a pattern often disrupted in cancer cells. Truncated O-glycans are found in most epithelial cancers including serous ovarian, uterine, stomach, colon and pancreatic cancers, and are associated with malignant transformation, cancer growth, metastatic ability, and modulation of immune response toward a tolerogenic TME (10, 11, 30).

A study demonstrated that human endometrial cancer cell lines expressing fucosylated and extended core 1 O‐glycans have high metastatic potency and that this feature can be due to the aberrant expression of the glycoprotein CD166 (31). CD166 is a cell adhesion molecule involved in the process of metastasis in multiple types of cancer (32). Treatment of these cancer cells with the human mAb HMMC-1, which recognizes O-glycans expressed on CD166, showed that this antibody not only can mediate the killing of these cells *in vitro* via ADCC and CDC, but that the binding to O-glycans attached to CD166 resulted in the arrest the cell cycle in the G1 phase, inducing cyclin-dependent kinase inhibitors p16 and p21 (31).

In this study, we demonstrated that NEO-201 can recognize human endometrial cancer cells expressing its target antigen either by flow cytometry or IHC. In flow cytometry we found two human endometrial cancer cell lines NEO-201 positive (ACI158 and ACI80), while in IHC only ACI158 was NEO-201 positive. This discrepancy can be due to differences in staining procedure, sample preparation, and sensitivity between flow cytometry and IHC. It is important to note that flow cytometry is often more sensitive than IHC for detecting cells with antigens low-expressed on their surface and this can, in part, explain the fact that ACI80 did not react with NEO-201 in IHC.

NEO-201 is a humanized IgG1 mAb which binds specifically to core 1 and/or extended core 1 O-glycans expressed by human cancer cells, non-cancerous CD15^+^ granulocytes and immunosuppressive cells, such as Tregs and gMDSCs (19, 23) and can mediate the killing of its target cells through several mechanisms of action, including ADCC (19). Regarding the employment of NEO-201 as a cancer drug for the treatment of gynecological cancers, a previous study demonstrated that NEO-201 reacts with different human ovarian cancer subtypes in IHC, with the strongest reactivity detected against ovarian mucinous adenocarcinoma, and that NEO-201 can mediate ADCC against the ovarian cancer cell line OV-90 *in vitro*, using NK cells as effectors (22). The ability of NEO-201 to mediate ADCC against OV-90 cells was also proved *in vivo* using murine models bearing ovarian cancer derived from OV-90 cell line. Mice treated with NEO-201 in combination with PBMCs as effectors showed a significant reduction of the tumor growth and a longer survival compared to mice treated with NEO-201 or PBMCs alone (22).

In this study we performed an ADCC assay *in vitro* to evaluate if NEO-201 has a better ADCC activity with NK cells or PBMCs as effectors against human endometrial and ovarian cancer cell lines expressing its target antigen. Results from this study showed that NEO-201 mediates ADCC against gynecological cancers expressing NEO-201 target antigen with NK cells as effector cells and that ADCC mediated by NEO-201 is dependent on CD16 engagement.

These results are in line with other studies that demonstrated that NEO-201 uses NK cells as main effectors to mediate ADCC against human cancer cell lines expressing its target antigen, including pancreatic cancer cell lines (CFPAC-1 and ASPC-1), the AML cell line HL-60, the breast cancer cell line ZR-75-1, lung cancer cell lines (H520 and HCC827) (20, 23, 27).

The anti-antitumor activity of NK cells, either through direct killing or via ADCC mediated by mAbs, can be enhanced by modulatory cytokines, such as IL-15 (33). A previous study demonstrated that the ADCC mediated by NEO-201 against cancer cells expressing its target antigen can be enhanced *in vitro* stimulating human purified NK cells with the IL-15 superagonist complex N-803 (formerly known as ALT-803) (27). In this study, we evaluated the capacity of a recombinant human IL-15 to enhance NEO-201-mediated ADCC *in vitro* against human endometrial and ovarian cancer cell lines comparing IL-15-stimulated PBMCs vs purified NK cells as effector cells. We showed that IL-15 enhanced ADCC mediated by NEO-201 in a statistically significant manner only when NK cells were used as effector cells against the endometrial cancer cell line ACI158 (3/3 healthy donors) and the ovarian cancer cell line OV-90 (1/3 healthy donors). These data provide evidence that IL-15 can enhance ADCC mediated by NEO-201 in gynecological cancers expressing NEO-201 target antigen.

To confirm *in vivo* that IL-15 enhances the ADCC mediated by NEO-201 with NK cells as effectors, we used a murine model bearing human ovarian cancer derived by OV-90 cell line to assess if the addition of IL-15 to NEO-201 can prolong the survival. The human ovarian cancer cell line OV-90 is reactive with NEO-201 in both IHC and flow cytometry and sensitive to NEO-201-mediated ADCC. This cell line recapitulates human ovarian cancer in murine models, and a previous study showed that this cell line carries missense mutations of the zinc-finger protein ZNF141 and major histocompatibility complex HLA-DRB5 genes. The role of these genes in modulating aberrant expression of O-glycans in cancer cells is not yet fully elucidated (22). Mice treated with NK cells + NEO-201 + IL-15 showed the longest median survival compared to all other groups, with a statistically significant difference in median survival observed comparing mice treated with NK cells + NEO-201 + IL-15 vs mice treated with NK cells + IL-15. Although *in vitro* we observed that IL-15 enhanced ADCC mediated by NEO-201 in a statistically significant manner against OV-90 cell line only when NK cells were used as effector cells in 1/3 healthy donors, the difference in median survival between mice treated with NK cells + NEO-201 + IL-15 and mice treated with NK cells + NEO-201 was not statistically significant. These observations show that IL-15 has a modest effect in prolonging the survival of mice bearing human ovarian cancer treated with NEO-201.

This phenomenon can be due to several factors: 1) This model was designed to follow survival rather than measure the tumor volume. The combination of IL-15+ NEO-201+ NK cells may have had a better effect on reducing tumor volume compared to NK cells + NEO-201 as observed *in vitro* and a more modest effect on survival; 2) we followed the survival until 120 days of treatment. For example, at 120 days 8/9 mice in the group treated with NK cells + NEO-201 + IL-15 were still alive, compared to 7/9 in the group treated with NK cells + NEO-201. Following survival longer than 120 days could have resulted in a statistically significant difference in median survival between those groups; 3) the half-life of the recombinant human IL-15 used in this study was too short to achieve results on long term effects on survival and the mice may have needed to be rechallenged with IL-15 more frequently. One of the main drawbacks in using IL-15 systemically *in vivo* is its half-life and limited bioactivity. In addition, a continuous boost of NK cells with IL-15 can result in their exhaustion and impairment of antitumor activity (34, 35), making repeated IL-15 administration an ineffective strategy to enhance the efficacy of NEO-201 when NK cells are used as effectors; 4) the lifespan of NK cells is limited *in vivo.* The employment of engineered NK cells in combination with NEO-201 could achieve better efficacy for the treatment of gynecological cancers.

Further studies, using different murine models of gynecological cancers, such as murine models for endometrial cancer, and IL-15/IL-15 receptor α complexes with a longer *in vivo* half-life and efficacy, are needed to achieve a better response in terms of survival in mice bearing gynecological cancers (35–37). In this regard, IL-15-secreting CAR-NK cells may be a promising source of engineered NK cells to be combined with NEO-201 to enhance its efficacy and overcome the limitations of short half-life of IL-15 and limited lifespan of NK cells (38).

It is also important to note that the human endometrial cancer cell line ACI158 (the only cell line reactive with NEO-201 in both IHC and flow cytometry and sensitive to NEO-201-mediated ADCC) did not carry any missense mutation, gene amplification, frame shift deletion or insertion of genes considered as variants of interest.

On the contrary, in all the other endometrial cancer cell lines not reactive with NEO-201 in IHC we observed different mutations in variants of interest.

Since the aberrant expression of O-glycans is one of the mechanisms involved in the progression of gynecological cancers (7, 8), further studies are needed to have a deep understanding if specific alterations of the status of specific genes associated with cancer formation and progression can be involved in the modulation of expression of O-glycans recognized by NEO-201. This information will guide clinicians to select the specific type of gynecological cancer that can benefit most from the treatment with NEO-201 alone or in combination with IL-15. Data provided in this study and existing data from previous studies on the safety and efficacy of NEO-201 in clinical trials for the treatment of several types of solid tumors, make NEO-201 a promising candidate for the treatment of gynecological cancers (39, 40). In addition, the fact that IL-15 enhanced ADCC activity of NEO-201 only when NK were used as effectors supports the hypothesis of combining NEO-201 and IL-15 with NK cell therapy to enhance the efficacy of NEO-201 for the treatment of solid tumors expressing O-glycans recognized by NEO-201.

## Data Availability

All data generated or analyzed during this study are included in this published article and in its supplementary information files. Raw data for this study were generated at the following institutions: Women’s Malignancies Branch, Center for Cancer Research, National Cancer Institute, National Institutes of Health, Bethesda, MD, USA; Molecular Histopathology Laboratory, Laboratory Animal Sciences Program, Frederick National Laboratory for Cancer Research, Frederick, MD, USA; Center for Cancer Research Collaborative Bioinformatics Resource (CCBR), National Cancer Institute, National Institutes of Health, Bethesda, MD, USA. Derived data supporting the findings of this study are available from the corresponding author upon request.

## Supplementary material

Supplementary Table 1 Tumor H-score of human endometrial cancer cell lines and colon cancer tissue stained with NEO-201 in IHC. For quantification of tumor expression of NEO-201 target antigen, tissues and cancer cell lines were binned based on staining intensity as negative, 1+ (mild staining; tumor tissues or cancer cell lines with a complete staining of the membrane in <10% of the sample analyzed), 2+ (moderate staining), 3+ (strong staining). Tissues and cancer cell lines with 2+ and 3+ staining intensity were defined as tumor tissues or cancer cell lines with a complete staining of the membrane in more than 10% of the sample analyzed. The % of positive cells was determined for each bin and the tumor H-score was calculated as follow: H-score = [1*(%1+) + 2*(%2+) + 3*(%3+)].

Supplementary Table 2 FIGO stage and type of uterine cancer of subjects with tissues analyzed in TMA. Table represents histology and FIGO stage of uterine cancer patients from whom tissues were derived from and used in the TMA. Types of uterine cancers included in the TMA were endometrioid G1 (118) and G2 (67). Abbreviations: FIGO = International Federation of Gynecology and Obstetrics.

Supplementary Table 3 Comparison of mice survival by log-rank (Mantel-Cox) test. The statistically significant difference in median survival between two treatments is reported in bold. NS: Not Significant.

Supplementary Figure 1 Representative flow cytometry plot for each human cancer cell line. The figure shows flow cytometry plots (unstained and NEO-201 stained cells) for each human endometrial and ovarian cancer cell line tested for NEO-201 binding. For each cell line, the percentage of unstained cells and NEO-201 stained cells with the MFI is also reported. NEO-201 positivity was defined as % positive ≥10%. NEO-201 positive cell lines appear in bold text.

Supplementary Figure 2 ADCC assay to evaluate the ability of IL-15 to enhance NEO-201-mediated ADCC at different concentrations in different human carcinoma cell lines. NEO-201 and human IgG1 (isotype control) were used at a concentration of 10 µg/mL. PBMCs and purified NK cells were treated with IL-15 (2, 1, 0.5 and 0.25 ng/mL) or vehicle control for 48 hours before being used as effector cells at the indicated E:T ratios. Results are presented as mean ± S.E.M. from two replicate wells. Asterisks denote statistical significance (two-way ANOVA). *p < 0.05; **p < 0.01; ***p < 0.001.

Supplementary Figure 3 ADCC assay to test the capacity of one healthy donor’s PBMCs and NK cells to mediate ADCC against OV-90 cell line in presence of NEO-201. Human ovarian carcinoma cell line OV-90 was used as target cell in the presence of 10 µg/mL of NEO-201 or human IgG1 (isotype control) in the ADCC assay. PBMCs and purified NK cells were used as effector cells at the indicated E:T ratios. Results are presented as mean ± S.E.M. from two replicate wells. Asterisks denote statistical significance (two-way ANOVA). ***p < 0.001.
