# Supplementary material for: IL 15 enhances preclinical efficacy of anti-core 1 O-glycans monoclonal antibody NEO-201 against human endometrial and ovarian cancer
Source: Front Immunol. 2026 Feb 24;17:1652596. doi: 10.3389/fimmu.2026.1652596 (PMC12971406; doi:10.3389/fimmu.2026.1652596)
Supplement: Supplementary Table 1 — Tumor H-score of human endometrial cancer cell lines and colon cancer tissue stained with NEO-201 in IHC. For quantification of tumor expression of NEO-201 target antigen, tissues and cancer cell lines were binned based on staining intensity as negative, 1+ (mild staining; tumor tissues or cancer cell lines with a complete staining of the membrane in <10% of the sample analyzed), 2+ (moderate staining), 3+ (strong staining). Tissues and cancer cell lines with 2+ and 3+ staining intensity were defined as tumor tissues or cancer cell lines with a complete staining of the membrane in more than 10% of the sample analyzed. The % of positive cells was determined for each bin and the tumor H-score was calculated as follow: H-score = [1*(%1+) + 2*(%2+) + 3*(%3+)]. [file Table1.docx]

| Cell Line | Tumor Type | % NEO-201 Positive | NEO-201 H-score |
| --- | --- | --- | --- |
| ACI126 | Endometrial serous adenocarcinoma | 0.00 | 0.00 |
| ACI158 | Endometrial serous adenocarcinoma | 18.30 | 20.95 |
| ACI52 | Endometrial adenocarcinoma | 0.12 | 0.12 |
| ACI80 | Endometrial adenocarcinoma | 0.80 | 0.81 |
| ACI98 | Endometrial undifferentiated carcinoma | 0.00 | 0.00 |
| ARK-1 | Endometrial serous adenocarcinoma | 0.04 | 0.04 |
| ARK-2 | Endometrial serous adenocarcinoma | 1.52 | 1.52 |
| EC1 | Endometrial adenocarcinoma | 0.02 | 0.02 |
| EC2 | Endometrial clear cell carcinoma | 0.07 | 0.07 |
| **Colon cancer tissue (positive control)** |  | **91.49** | **126.66** |
