# Supplementary material for: IL 15 enhances preclinical efficacy of anti-core 1 O-glycans monoclonal antibody NEO-201 against human endometrial and ovarian cancer
Source: Front Immunol. 2026 Feb 24;17:1652596. doi: 10.3389/fimmu.2026.1652596 (PMC12971406; doi:10.3389/fimmu.2026.1652596)
Supplement: Supplementary Table 2 — FIGO stage and type of uterine cancer of subjects with tissues analyzed in TMA. Table represents histology and FIGO stage of uterine cancer patients from whom tissues were derived from and used in the TMA. Types of uterine cancers included in the TMA were endometrioid G1 (118) and G2 (67). Abbreviations: FIGO = International Federation of Gynecology and Obstetrics. [file Table2.docx]

| **Type of uterine** c**ancer** | **Number of subjects (%)** | **FIGO stage** | Number of subjects (%) | | | |
| --- | --- | --- | --- | --- | --- | --- |
| Endometrioid G1 | 118 (63.8%) | IA | 140 (77.8%) | | | |
| Endometrioid G2 | 67 (36.2%) | IB | 40 (22.2%) | | | |
|  |  |  |  | | | |
|  |  |  | |  |  |  |

Abbreviations: FIGO = International Federation of Gynecology and Obstetrics
