# Supplementary material for: IL 15 enhances preclinical efficacy of anti-core 1 O-glycans monoclonal antibody NEO-201 against human endometrial and ovarian cancer
Source: Front Immunol. 2026 Feb 24;17:1652596. doi: 10.3389/fimmu.2026.1652596 (PMC12971406; doi:10.3389/fimmu.2026.1652596)
Supplement: Supplementary Table 3 — Comparison of mice survival by log-rank (Mantel-Cox) test. The statistically significant difference in median survival between two treatments is reported in bold. NS: Not Significant. [file Table3.docx]

| **Treatment** | **Hazard ratio** | **95% hazard ratio confidence limitis** | | ***p* value** | **Median survival (days)** |
| --- | --- | --- | --- | --- | --- |
|  |  | **lower** | **upper** |  |  |
|  | | | | | |
| NK + NEO-201 + IL-15 vs  NK + NEO-201 | 2.92 | 0.41 | 20.75 | NS | Undefined |
| **NK + NEO-201 + IL-15 vs**  **NK + IL-15** | **8.34** | **1.86** | **37.47** | **0.0122** | **Undefined vs 100** |
| NK + NEO-201 vs NK + IL-15 | 0.34 | 0.09 | 1.29 | NS | Undefined vs 100 |

**Log-rank test**
