# Supplementary figures and images for: IL 15 enhances preclinical efficacy of anti-core 1 O-glycans monoclonal antibody NEO-201 against human endometrial and ovarian cancer
Source: Front Immunol. 2026 Feb 24;17:1652596. doi: 10.3389/fimmu.2026.1652596 (PMC12971406; doi:10.3389/fimmu.2026.1652596)

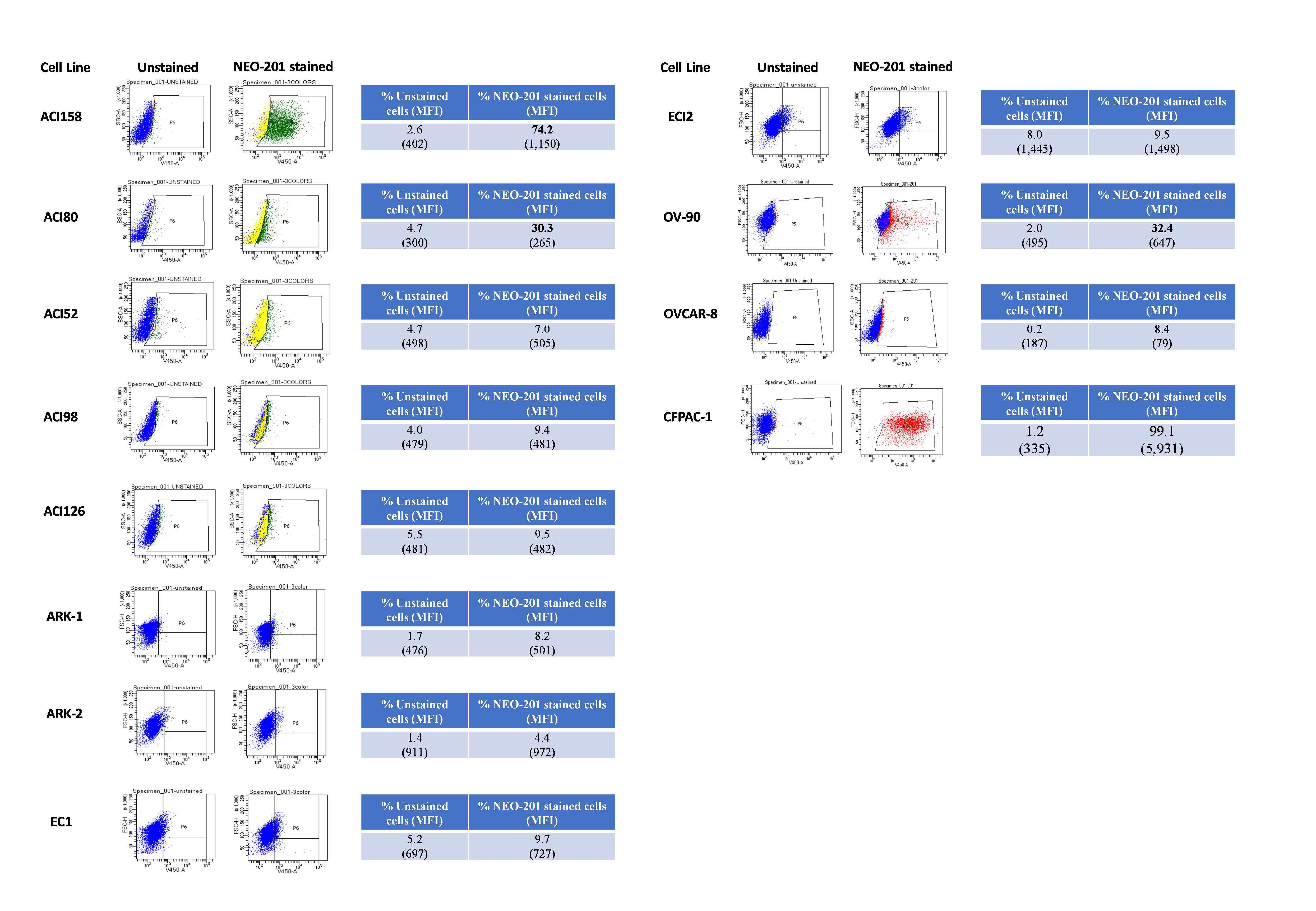

Supplement: Supplementary Figure 1 — Representative flow cytometry plot for each human cancer cell line. The figure shows flow cytometry plots (unstained and NEO-201 stained cells) for each human endometrial and ovarian cancer cell line tested for NEO-201 binding. For each cell line, the percentage of unstained cells and NEO-201 stained cells with the MFI is also reported. NEO-201 positivity was defined as % positive ≥10%. NEO-201 positive cell lines appear in bold text. [file Image1.tif]

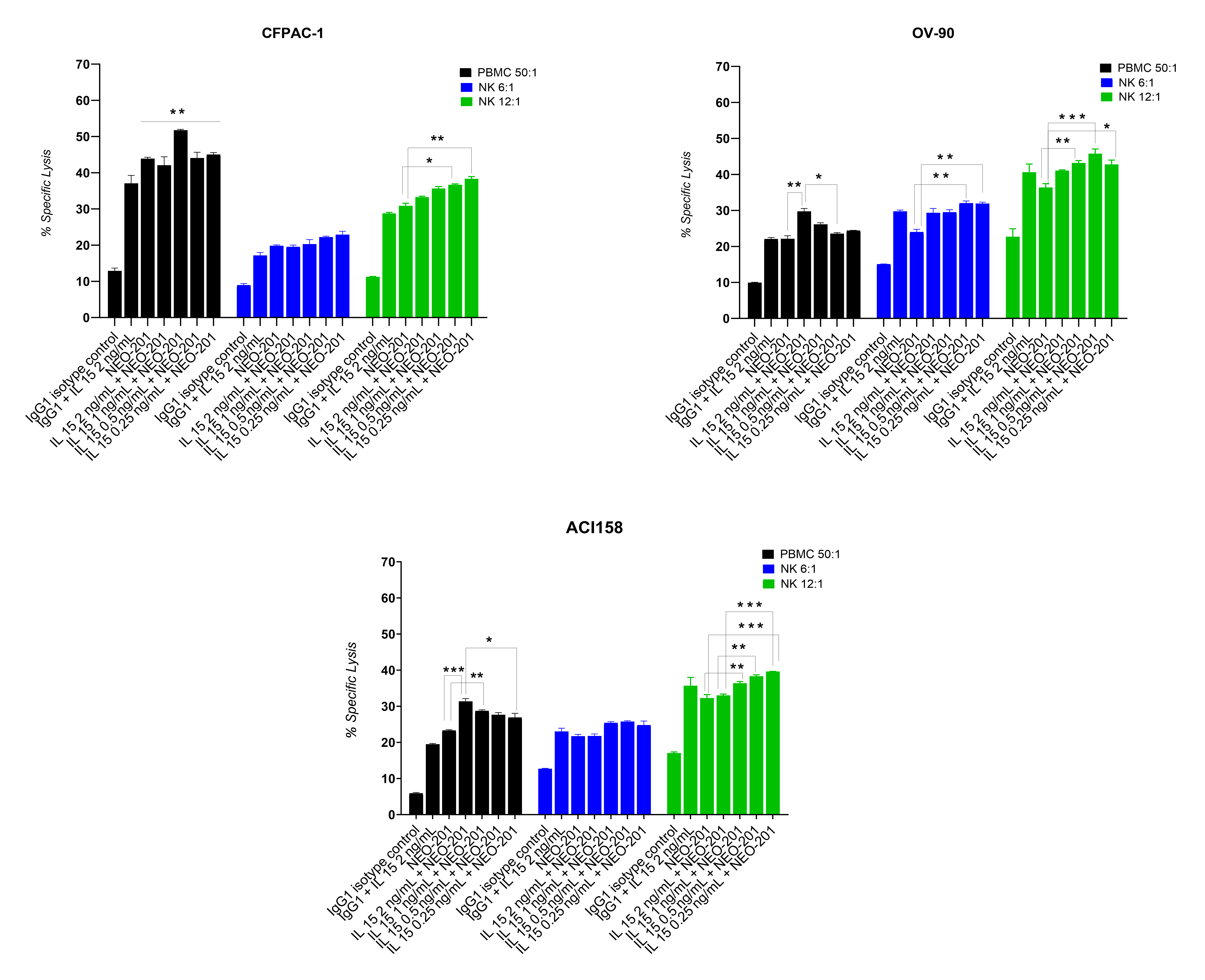

Supplement: Supplementary Figure 2 — ADCC assay to evaluate the ability of IL-15 to enhance NEO-201-mediated ADCC at different concentrations in different human carcinoma cell lines. NEO-201 and human IgG1 (isotype control) were used at a concentration of 10 µg/mL. PBMCs and purified NK cells were treated with IL-15 (2, 1, 0.5 and 0.25 ng/mL) or vehicle control for 48 hours before being used as effector cells at the indicated E:T ratios. Results are presented as mean ± S.E.M. from two replicate wells. Asterisks denote statistical significance (two-way ANOVA). *p < 0.05; **p < 0.01; ***p < 0.001. [file Image2.tif]

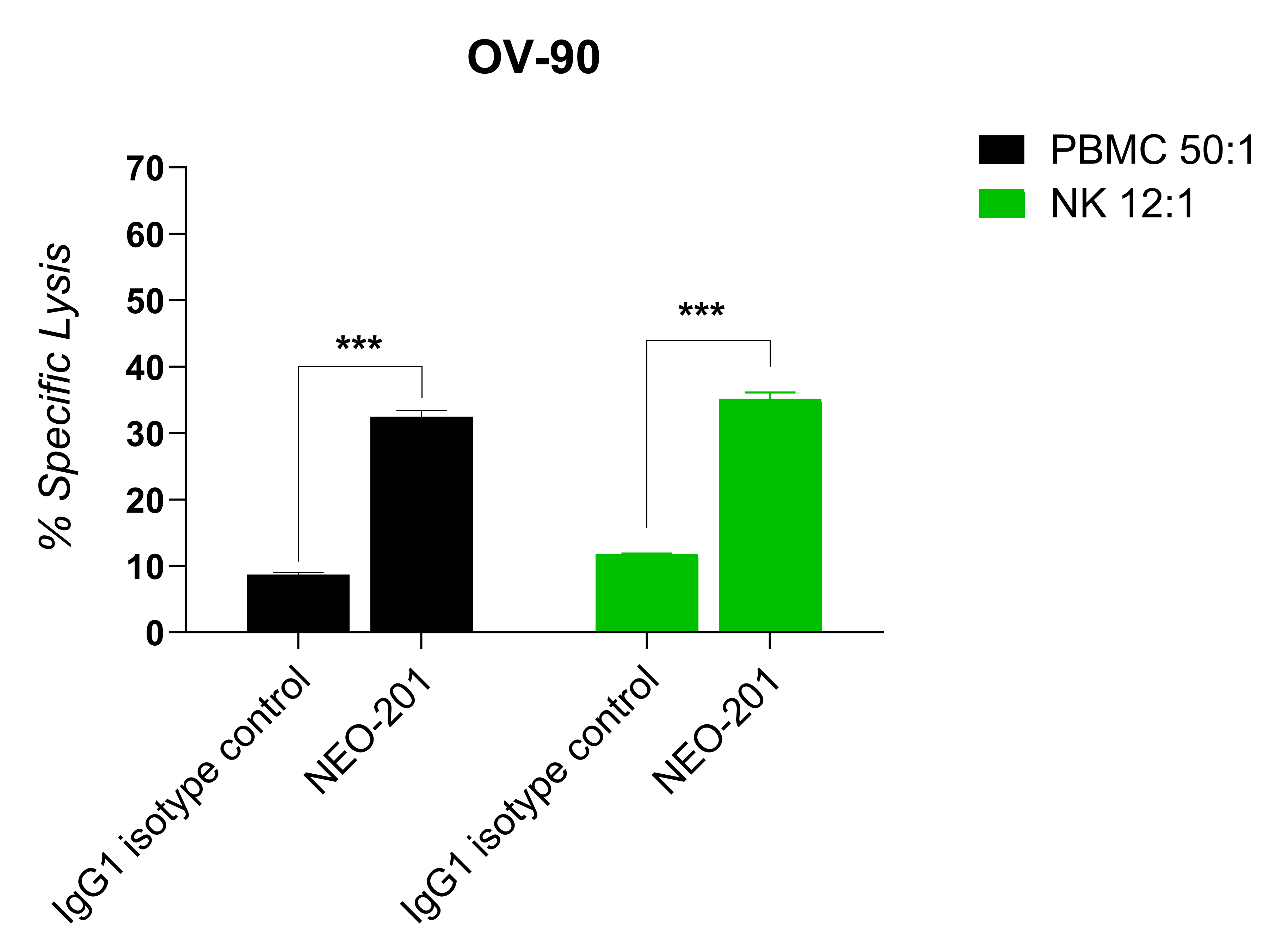

Supplement: Supplementary Figure 3 — ADCC assay to test the capacity of one healthy donor’s PBMCs and NK cells to mediate ADCC against OV-90 cell line in presence of NEO-201. Human ovarian carcinoma cell line OV-90 was used as target cell in the presence of 10 µg/mL of NEO-201 or human IgG1 (isotype control) in the ADCC assay. PBMCs and purified NK cells were used as effector cells at the indicated E:T ratios. Results are presented as mean ± S.E.M. from two replicate wells. Asterisks denote statistical significance (two-way ANOVA). ***p < 0.001. [file Image3.tif]
